# Longtime soaking of high concentration tranexamic acid in total hip arthroplasty: A prospective randomized controlled trial in 224 patients

**DOI:** 10.12669/pjms.316.8465

**Published:** 2015

**Authors:** Xingming Xu, Xiaofeng Li, Wei Liu, Zhenyu Wang

**Affiliations:** 1 Xingming Xu, MD. Medical College of Nanchang University, Nanchang University, No.461, Bayi Street, 330000 Nanchang, China; 2 Xiaofeng Li, MD. Dept. of Orthopaedics, The First Affiliated Hospital of Nanchang University, Nanchang University, No.17, Yongwai Street, 330006 Nanchang, China; 3 Wei Liu, MD. Medical College of Nanchang University, Nanchang University, No.461, Bayi Street, 330000 Nanchang, China; 4 Zhenyu Wang, MD. Medical College of Nanchang University, Nanchang University, No.461, Bayi Street, 330000 Nanchang, China

**Keywords:** Tranexamic Acid, Total hip arthroplasty, Intra-articular soaking, Total blood loss

## Abstract

**Objectives:**

To evaluate the efficacy and safety of intra-articular Soaking of high concentration Tranexamic Acid (TXA) in total hip arthroplasty.

**Methods:**

From March 2013 to March 2014, 224 patients who underwent unilateral primary THA in our hospital was enrolled in this randomized, prospective double-blinded study. The patients were allocated into two groups according to intra-articular solution received: Intra-articular soaking of TXA group, Control group (physiologic saline). The solution was injected from intermuscular space following fixation of the implants and closure of articular capsule, short external rotators. Total blood loss, total volume of drainage and transfusion were recorded. Postoperative deep vein thrombosis and other complications was also measured.

**Results:**

The mean total blood loss was 730±296 ml in intra-articular soaking of TXA group compared with 1048±295ml in control group (P<0.05). The postoperative mean total volume of drainage was 93±50 mL in intra-articular soaking of TXA group versus 312±136 mL in control group.22 patients (19.8%, control) and 6 patients (5.3%, Intra-articular soaking of TXA) required transfusion (P=0.001). Postoperative deep vein thrombosis and other complications were no statistical significance between the two groups.

**Conclusions:**

Intra-articular soaking of high concentration TXA with 2-hour clamping drain can reduce the total blood loss and transfusion rates in primary THA without significant increase in postoperative thrombotic complications.

## INTRODUCTION

In recent years, with total hip arthroplasty in a large number of clinical application, artificial total hip arthroplasty can help the femoral neck fracture, aseptic necrosis of femoral head, congenital developmental dysplasia of the hip and rheumatoid arthritis patients with hip pain relief and reconstruction hip function. However, THA is associated with considerable perioperative blood loss and required transfusion subsequently.^1^,^2^ The patients who need surgery tend to be older people and a large amount of blood loss is easy to aggravate underlying disease of the old patients. Moreover allogenic transfusion may carry the potential risks of adverse immunological reactions, intravascular haemolysis, transfusion-induced coagulopathy, disease transmission, renal failure, and even increased mortality.^3^,^4^ Therefore, a reduction in the bleeding and allogenic transfusion of THA has become an increasingly concerned topic.

Tranexamic acid (TXA), a synthetic derivative of lysine, prevent fibrinolysis by reversible blockade of the lysine-binding sites of plasminogen, thereby achieving the goal of local hemostasis and reducing bleeding.^5^-^6^ Several studies have shown that the effectiveness of intravenous tranexamic acid application in reducing postoperative blood loss in THA and TKA.^7^-^9^ However, the risk of systemic thrombotic events have been taken into consideration and the systemic administration of tranexamic acid which is inconsistent with the concept of postoperative anti-coagulation therapy after joint replacement. Therefore more and more Scholars begin to transfer their attentions to topical use of TXA. One of the methods, Intra-articular administration of tranexamic acid has shown the safety and effectiveness in TKA.^10^-^12^ So far, there are few studies^13^-^14^ about intra-articular administration of tranexamic acid in THA. The purpose of this prospective randomized trial was to determine the efficacy and safety of intra-articular soaking of high concentration TXA in total hip arthroplasty.

## METHODS

This randomized, prospective double-blinded study was performed on patients who underwent unilateral primary THA in our hospital between March 2013 and March 2014. Prior to starting this trial, institutional review board approval was obtained, and formed consent was obtained from all patients preoperatively.

The inclusion criteria were the patients diagnosed as femoral neck fracture, femoral head necrosis, osteoarthritis, and underwent unilateral primary cementless THA. The exclusion criteria were patients with hemorrhagic blood diseases; pre-operative use of anticoagulant treatment within 7 days; allergy to tranexamic acid; hemoglobin (Hb) <90 g/L; contra-indication for TXA use; thrombotic disorder.

**Fig.1:**
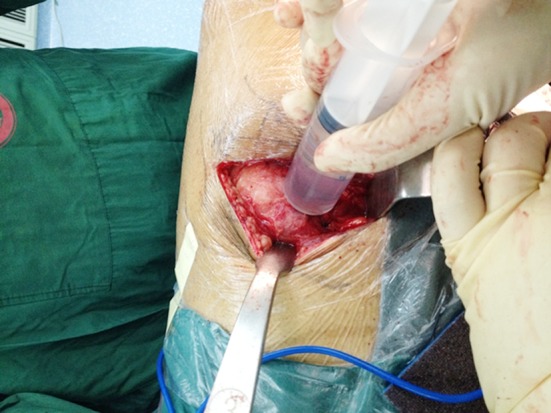
Inject 40 mL sterile saline with 3g of tranexamic acid into hip articular cavity from intermuscular space following fixation of the implants and closure of articular capsule, short external rotators.

Two hundred twenty four patients were eligible for inclusion in the study. Sealed envelopes with number in container was used. After articular capsule and short external rotators (Including piriformis, superior gemellus, inferior gemellus, quadratus femoris) were reattached to the posterior border of the greater trochanter by several bone holes after replacement, one of our researchers, who did not involve in the operation and in evaluation of the results, was responsible for opening these envelopes and preparing the study medication. Then all patients were allocated to one of two groups; control group or experimental group. The experimental group contained 113 patients injected 3g of tranexamic acid in 40 mL of sterile saline into hip articular cavity from intermuscular space following fixation of the implants and closure of articular capsule, short external rotators (Fig.1), whereas the control group consisted of 111 patients who received 40 mL of sterile saline in the same way. Patients, surgeons and nurses are blinded to the whole process.

All the surgeries were done under continuous lumbar epidural anesthesia. Surgeries were performed by the same surgeon and the whole operations were completed through the posterolateral hip approach. The prosthesis was a cementless acetabular cup and a cementless femoral stem. According to envelopes result, the experimental group injected 3g of tranexamic acid in 40 mL of sterile saline into hip articular cavity from intermuscular space following fixation of the implants and closure of articular capsule, short external rotators, whereas the control group received 40 mL of sterile saline in the same way. One drain was placed and clamped for two hours in both groups respectively. The drainage was removed in 48 hours after the operation. For venous thromboembolism prophylaxis, all patients were given rivaroxaban 10mg orally once daily for 35 days.

Demographic data were collected before the operation included age, gender, height, weight, levels of Hb and hematocrit (Hct), fibrinogen (FIB), prothrombin time (PT) and activated partial thromboplastin time (APPT) were measured before and after the operation.

The primary outcome measured were the rate of deep vein thrombosis (DVT) and pulmonary embolism (PE), transfusion rate, difference between the preoperative hemoglobin and the lowest postoperative hemoglobin during the hospital stay. Every patient was given ultrasound examination routinely for screening DVT and CT pulmonary angiography for PE in the fifth day after operation. Symptomatic DVT and Pulmonary embolism (PE) were observed continuously by follow-up for six months. Patients received blood transfusions according to the following protocol: (1) patients with Hb levels less than 70 g/L; (2) patients with Hb levels more than 70 g/L received a transfusion determined by the mental state of the patients. The secondary outcomes were total volume of drainage, intraoperative blood loss, total blood loss and other perioperative complications. The staff who evaluated the results was blinded to the allocation. Blood volume of each patient was calculated according to the formula of Nadler et al.^15^ Total blood loss was calculated according to the formula of Gross.^16^

Blood volume = k1×height (m) + k2×weight (kg) + k3; k1 = 0.3669, k2 = 0.03219, and k3 = 0.6041 for men; and k1 = 0.3561, k2 = 0.03308, and k3 = 0.1833 for women

Total blood loss=Blood volume×(HO-HF)/Hav; HO=the preoperative hematocrit, HF=the postoprative hematocrit, Hav=the average of preoperative hematocrit and postoprative hematocrit.

All the quantitative datas have been conducted normality test and the statistical significance between the two groups (control group, intra-articular injection of TXA group) were determined using two-sample Student-t test. Pearson chi-square test or Fisher exact test was used to analyze the qualitative variable. We used SPSS for Windows version 19.0. A p value <0.05 was considered significant.

## RESULTS

From March 2013 to March 2014, there were 304 patients scheduled to have primary unilateral THA in The First Affiliated Hospital of Nanchang University. 67 patients were excluded after prescreening by inclusion and exclusion criteria, 13 patients declined participation. Thus, the data of 224 patients were analyzed at last.

The characteristics of the patients were shown in Table-I. There were no statistical significance between the two groups. This included no differences in baseline age, gender, body mass index, Preoperative laboratory index, intraoperative blood loss and Postoperative complications (including deep vein thrombosis, pulmonary embolism and Wound infection).

**Table-I:** Characteristics of the patients.

	Intra-articular soaking of TXA group		Control group
	(n=113) Mean±SD	(n=111) Mean±SD	P-value
*Demographic characteristics*			
Age(years)	67.5±10.7	67.4±8.8	0.943
Gender: male/female	46/67	45/66	0.980
Body mass index (kg/m^2^)	22.0±3.2	22.2±2.7	0.654
*Preoperative laboratory indexes*
Prothrombin time (seconds)	10.6±0.9	10.6±1.2	0.845
Plasma fibrinogen (g/L)	3.0±0.6	2.9±0.5	0.328
APTT (seconds)	28.9±4.1	29.5±3.8	0.227
Intraoperative blood loss (ml)	163±41	174±63	0.133
*Postoperative complications*
Number of DVT	1	1	1.00
Number of PE	0	0	1.00
Wound infection	0	1	1.00

APTT: Activated Partial Thromboplastin Time. DVT: Deep Vein Thrombosis. PE: Pulmonary Embolism.

**Fig.2:**
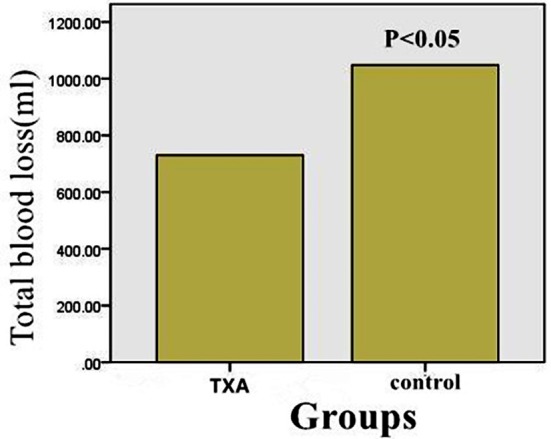
The histogram demonstrated that total blood loss in Intra-articular injection of TXA groupwas significantly less than control group.

**Fig.3:**
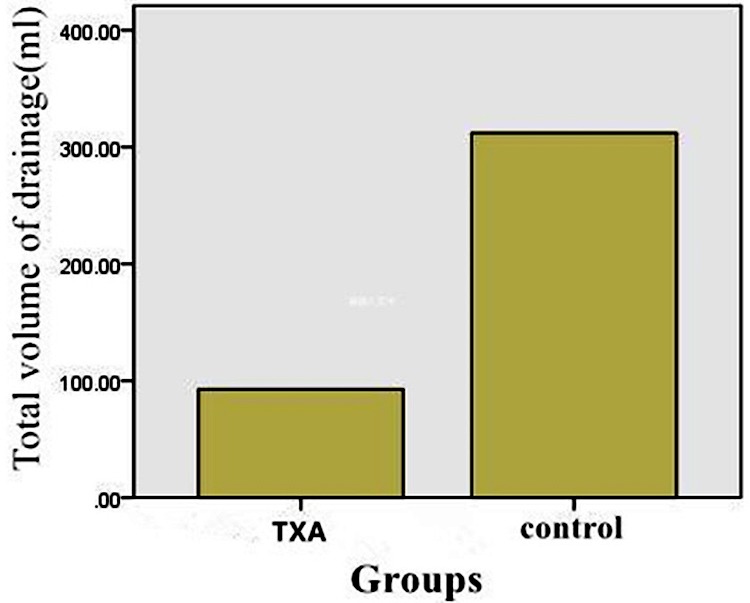
The histogram demonstrated that total volume of drainage in Intra-articular injection of TXA group was significantly less than control group.

The maximum decline between the preoperative hemoglobin and the lowest postoperative hemoglobin was significantly lower (P<0.05) in intra-articular soaking of TXA group than in control group (Table-II). The mean total blood loss was significantly less (P<0.05) in intra-articular soaking of TXA group (730±296 ml) than (1048±295ml) in control group (Table-II and Fig.2). The postoperative total volume of drainage was significantly less (P<0.05) in the patients who received intra-articular soaking of TXA (93±50 mL) versus (312±136 mL) in control group (Table-II and Fig.3). The blood transfusion rate has dropped considerably for intra-articular soaking of TXA group. Nearly twenty percent of patients who received placebo were transfused and 5.3% of patients who received TXA were transfused (Table-II).

**Table-II:** The major outcomes of two groups.

	Intra-articular soaking of TXA group		Control group
	(n=113) Mean±SD	(n=111) Mean±SD	P-value
Hemoglobin decline(g/L)	27.8±9.6	39.3±12.9	0.00*
Total blood loss(ml)	730±296	1048±295	0.00*
Total volume of drainage(ml)	93±50	312±136	0.00*
Number of blood transfusion	6(5.3%)	22(19.8%)	0.001*

* Significant difference.

## DISCUSSION

TXA is becoming increasingly popular in clinical trials for reducing operative blood loss.^17^-^19^ As shown in this randomized double-blind controlled trial, a high concentration intra-articular soaking of TXA could conspicuously reduce total volume of drainage, total blood loss. Meanwhile, we have found that intra-articular soaking of TXA could effectively reducing transfusions from 19.8% to 5.3% without increasing the risk of DVT and other complications in THA. Therefore, intra-articular soaking of high concentration TXA was effective and safe for THA.

In recent studies of topical or intra-articular administration of TXA in THA were still deficient. Konig and Yue C et al.^13^,^20^ topical administration of TXA could significantly reduce bleeding and transfusions in primary THA, but the process is a bit more complex and the method increased the duration of surgery by about six minutes, which might increase the risk of infection.^21^ Martin et al^22^ topical application of 2g TXA in total knee and hip arthroplasty resulted in a significant decline in blood loss. However, a larger sample size is needed to be more persuasive (only 53 hips). This large sample study of intra-articular soaking of TXA in THA has its advantage: it is simple to operate; tranexamic acid into hip articular cavity from intermuscular space directly target the site of bleeding; tranexamic acid have enough time and concentration thoroughly exposed to the bleeding points. The reason we used intra-articular soaking of TXA is that a large number of blood loss came from acetabulum and femoral canal. Intra-articular soaking of tranexamic acid offers an innovative therapeutic approach for decreasing total blood loss and transfusions rate. Therefore, we could conclude that intra-articular soaking of TXA was satisfactory in THA.

Tranexamic acid is antifibrinolytic agent which has been confirmed that intravenous TXA could effectively reduce bleeding and transfusion rates in THA.^7^,^22^ However, the risk of systemic thrombotic events must be taken into consideration. The results of our study are satisfactory compared with those of previous IV-TXA studies in THA patients. This confirmed that intra-articular soaking of TXA can reduce the total blood loss and transfusion rates effectively. Compared with IV-TXA, a intra-articular application has the advantages of easy to administer, providing a maximum concentration of TXA at the bleeding site, and was associated with little or no systemic absorption of the TXA.^23^ This study demonstrated no significant difference in thrombo-embolism events between intra-articular TXA group and control group. Therefore, we firmly believe that intra-articular TXA is safe in THA.

The present study has some limitations: First, the duration of follow-up time was too short. In addition, we don’t know exactly how much TXA has permeated through articular tissue surface to the systemic circulation since serum concentrations of TXA were not measured. Last, we cannot be certain whether TXA has a negative effect on osseous integration of the implants or on joint wear.

## CONCLUSIONS

The present study demonstrated that intra-articular soaking of high concentration TXA with 2-hour clamping drain can reduce the total blood loss and transfusion rates in primary THA without significant increase in postoperative thrombotic complications. We firmly believe that intra-articular longtime soaking of high concentration TXA is effective and safe to patients.
